# Supplementary material for: ProteinShader: illustrative rendering of macromolecules
Source: BMC Struct Biol. 2009 Mar 30;9:19. doi: 10.1186/1472-6807-9-19 (PMC2672931; doi:10.1186/1472-6807-9-19)
Supplement: Additional file 1 — ProteinShader program without source code. This compressed file contains the complete ProteinShader program including associated libraries, but no source code. A README.txt file gives an overview of the ProteinShader distribution, and the index.html file in the help subdirectory has directions on getting started with the program as well as a set of tutorials. [file 1472-6807-9-19-S1.zip › ProteinShader-beta-0_9_4-binary/help/api/overview-frame.html]

Overview (ProteinShader API)


All Classes

Packages
  
org.proteinshader.graphics
  
org.proteinshader.graphics.adapter
  
org.proteinshader.graphics.displaylists
  
org.proteinshader.graphics.enums
  
org.proteinshader.graphics.exceptions
  
org.proteinshader.graphics.shader
  
org.proteinshader.graphics.textures
  
org.proteinshader.graphics.typography
  
org.proteinshader.graphics.utils
  
org.proteinshader.gui
  
org.proteinshader.gui.components
  
org.proteinshader.gui.components.controlpanel
  
org.proteinshader.gui.components.menubar
  
org.proteinshader.gui.enums
  
org.proteinshader.gui.exceptions
  
org.proteinshader.gui.listeners
  
org.proteinshader.gui.listeners.controlpanel
  
org.proteinshader.gui.listeners.menubar
  
org.proteinshader.gui.utils
  
org.proteinshader.gui.viewing
  
org.proteinshader.math
  
org.proteinshader.structure
  
org.proteinshader.structure.enums
  
org.proteinshader.structure.exceptions
  
org.proteinshader.structure.factory
  
org.proteinshader.structure.io
  
org.proteinshader.structure.io.demos
  
org.proteinshader.structure.io.enums
  
org.proteinshader.structure.io.exceptions
  
org.proteinshader.structure.io.filters
  
org.proteinshader.structure.sort
  
org.proteinshader.structure.visitor
  
org.proteinshader.structure.visitor.demos
  
org.proteinshader.structure.visitor.enums
  
org.proteinshader.structure.visitor.exceptions
  
org.proteinshader.structure.visitor.modifiers
